# Associations with rates of falls among home care clients in Ontario, Canada: a population-based, cross-sectional study

**DOI:** 10.1186/s12877-020-1483-6

**Published:** 2020-02-27

**Authors:** Derek R. Manis, Caitlin McArthur, Andrew P. Costa

**Affiliations:** 1 Centre for Health Economics and Policy Analysis, McMaster University, 1280 Main Street West, CRL-201, Hamilton, ON L8S 4K1 Canada; 2 Department of Health Research Methods, Evidence, and Impact, McMaster University, Hamilton, Canada; 3 Department of Medicine, McMaster University, Hamilton, Canada; 4 GERAS Centre for Aging Research, McMaster University, Hamilton, Canada

**Keywords:** Accidental falls, Home care, Older adults, Assistive devices, Canada

## Abstract

**Background:**

Accidental falls among older adults are a leading cause of injury-related hospitalizations. Reducing falls is an ongoing quality improvement priority for home care, given that many home care clients experience falls. In this study, we identify factors associated with the rate of falls among home care clients.

**Methods:**

We conducted a population-based, cross-sectional study using secondary data from the Hamilton, Niagara, Haldimand, and Brant health region of Ontario, Canada from January 1 – March 31, 2018. We captured person-level characteristics with falls from the Resident Assessment Instrument – Home Care (RAI-HC). Negative binomial regression was used to model the rate of falls.

**Results:**

Functional characteristics of home care clients had strong, statistically significant associations with the rate of falls. Declines in activities of daily living, assistive device use for locomotion indoors, polypharmacy, and health conditions, such as dizziness or lightheadedness, and parkinsonism, were associated with a higher rate of falls. Males who used assistive devices had a higher rate of falls compared to females; however, males with neurological and cardiovascular health conditions had a decrease in the rate of falls compared to females. Home care clients with parkinsonism who used a cane and took eight or more drugs had stronger associations with an increased rate of falls compared to those who do not have parkinsonism.

**Conclusions:**

Functional characteristics, polypharmacy, and health conditions are associated with increased rates of falls among home care clients. Home care clients who are at a greater risk of falls may require environmental adjustments in their home to reduce or eliminate the possibility of falling.

## Introduction

Accidental falls are the predominant cause of all injury-related hospitalizations among older adults in Canada [1]. Accidental falls also adversely affect mental health, resulting in decreased independence and autonomy, and increased fear of falling, increased isolation, and depression [1–5]. Falls are also important predictors of older adults becoming institutionalized (i.e., admission to long-term care) [6]. In Ontario, home care services are predominately provided by the provincial government under its universal, public health insurance plan to support older adults in receiving the care services they need (e.g., nursing, physiotherapy, occupational therapy, social work, etc.) to remain in their home and community [7]. Reducing falls is an ongoing quality improvement priority for home care, given that many home care clients experience falls [8, 9].

Among home care clients in Ontario, Canada, risk factors for falls have been investigated among those with neurological conditions (i.e., dementia, parkinsonism) [10]. In addition, many chronic health conditions have been investigated in the context of home care and quality of life [11]. However, an explanatory investigation of multiple person-level characteristics with the rate of falls has not been conducted with recent data on home care clients. Person-level factors are important to investigate because these factors contribute to the development and implementation of strategies to prevent falls from occurring. Falls are also multifactorial, and so it is necessary to investigate multiple factors to identify clustering characteristics to classify patient groups that need more clinical focus to prevent falls. The rate of falls is important because it identifies the frequency of falling, which has implications for improving patient safety and quality of care in the home care setting.

Our study objective was to investigate the associations between person-level characteristics and the rate of falls among home care clients. We hypothesized that home care clients with polypharmacy, impaired cognition, declines in activities of daily living, and neurological disorders (i.e., Alzheimer’s, dementia, multiple sclerosis, and parkinsonism) were associated with falls in this population. Our secondary objective was to examine differences between males and females, and to examine differences between different high-risk subgroups (e.g., parkinsonism, etc.).

## Methods

### Study design, setting, and participants

We conducted a population-based, cross-sectional study in the Hamilton, Niagara, Haldimand, and Brant (HNHB) health region of Ontario, Canada. This region services over 1.4 million residents, of which 27% of the population is over the age of 65 [12]. Home care clients in the HNHB health region who received any type of home care assessment (e.g., initial assessment, follow-up, change in status, etc.) during the January 1, 2018 to March 31, 2018 period were included in our study. Only the first assessment for each home care client during the study period was included in our analysis.

### Data source

The Resident Assessment Instrument – Home Care (RAI-HC) is an assessment from interRAI for use with older adults who receive home care or are in a community-based setting. This assessment includes severity scales pertaining to cognitive, hearing, vision, mood and behavior patterns, and activities of daily living. It also captures health status (e.g., chronic health conditions and medications, preventive health measure, etc.), environmental assessment, and health service utilization [13]. The RAI-HC assessment is a valid and reliable instrument, has strong test/re-test reliability, and has been used in other studies investigating falls among home care clients [12, 14].

### Variables

The outcome variable, falls frequency, specifies the number of falls experienced by the home care client in the last 90 days. This variable is a count variable ranging from zero to nine, where nine or more falls is reported as nine. Predictor variables were selected using a combination of clinical judgment and an assessment of related literature on home care clients and adverse events in the home [1, 11, 15–18]. Demographic (e.g., age, sex, etc.), functional (e.g., cognitive skills, activities of daily living, assistive device use, etc.), and number of drugs taken and diagnoses (e.g., cardiovascular, neurological, musculoskeletal, etc.) were person-level characteristics included in the final model to determine the associations with falls among home care clients.

### Statistical methods

Sample size was calculated based on at least 20 events per predictor variable (*n* ≥ 820) [19]. Descriptive statistics (i.e., frequencies, percentages, and 95% confidence intervals) were calculated for all categorical variables in the model. No continuous variables were used; age and number of drugs taken by home care clients were transformed into categorical variables in 10-year and 2-medication intervals, respectively, to support clinical relevance, importance, and easier interpretation of the results. The outcome variable was not normally distributed; rather than transforming it, which would limit clinical interpretation of the results, negative binomial regression was used. Negative binomial regression was preferred to Poisson and Quasi-Poisson regression, given that the variance was greater than the mean.

Variable selection was performed by comparing Akaike Information Criterion between demographic and functional characteristics of home care clients and different groups of diagnoses (i.e., cardiovascular, neurological, musculoskeletal). An α = 0.05 was used for statistical significance for testing variables. Variance inflation factors were assessed for all variables. Interactions between sex and all predictors in the final model were assessed because of the differences that exist between males and females for various health conditions included in the final model (e.g., cardiovascular, musculoskeletal, etc.), and these interactions may additionally have an impact on associations with the rate of falls. Statistically significant interactions at α = 0.001 were reported. Outliers were assessed by examining standardized residuals with values greater than two. Data set processing was conducted in SAS Enterprise 9.4 (Cary, North Carolina, USA) and statistical analyses were conducted in R version 3.5.3 (Vienna, Austria) [20–29].

## Results

There were 10,586 home care clients in the HNHB health region who received an assessment during the January 1, 2018 to March 31, 2018 period (*n* = 10,586). The were no missing data, given that the RAI-HC is the basis for electronic medical records in the home care setting and assessment fields are mandatory [30]. The outcome variable, falls frequency in the last 90 days, was skewed to the right. Fifty-two per cent of the sample (*n =* 5481) did not experience a fall, whereas 40% (*n* = 4214) experienced one to three falls. Six per cent of the sample (*n* = 649) experienced four to eight falls, and 2 % of the sample (*n* = 242) experienced nine or more falls.

### Description of population-based sample

Table 1 describes our population-based sample of home care clients in the HNHB health region. Home care clients were predominately female (*n =* 6462, 61%), between the ages of 80–89 (*n =* 3920, 37%) and were widowed (*n* = 4363, 41%). Some home care clients had minimally impaired cognitive skills for daily decision-making (*n* = 2228, 21%), but most had declines in activities of daily living (*n =* 6915, 65%), used a walker or crutch for locomotion indoors (*n* = 4804, 45%), and were unable to go up and down the stairs (*n =* 6332, 60%). Over half of our sample took eight or more drugs (*n* = 6744, 64%), and many experienced dizziness or lightheadedness (*n* = 2795, 26%), edema (*n =* 3726, 35%), and shortness of breath (*n =* 3533, 33%).

**Table 1:** Characteristics of home care clients in HNHB health region, Jan 1 – Mar 31, 2018 (*n* = 10,586)

	% (*n*)	95% CI (%)
Demographic Characteristics		
Sex		
Male	39 (4160)	38, 40
Female	61 (6462)	60, 62
Age		
< 60	9 (949)	8.4, 9.5
60–69	12 (1246)	11, 12
70–79	22 (2301)	21, 23
80–89	37 (3920)	36, 38
90–99	20 (2114)	19, 21
≥ 100	0.5 (56)	0.4, 0.69
Marital Status		
Never married	8.6 (913)	8.1, 9.2
Married	38 (3972)	37, 38
Widowed	41 (4363)	40, 42
Separated	2.9 (302)	2.5, 3.2
Divorced	8.1 (861)	7.6, 8.7
Other	1.7 (175)	1.4, 1.9
Functional Characteristics		
Cognitive Skills for Daily Decision-Making		
Independent	33 (3465)	32, 34
Modified Independent	27 (2830)	26, 28
Minimally Impaired	21 (2228)	20, 22
Moderately Impaired	12 (1314)	12, 13
Severely Impaired	7.1 (749)	6.6, 7.6
Activities of Daily Living Decline	65 (6915)	64, 66
Primary Modes of Locomotion (Indoors)		
No Assistive Device	27 (2888)	26, 28
Cane	10 (1077)	9.6, 10.8
Walker/Crutch	45 (4804)	44, 46
Scooter	0.31 (33)	0.22, 0.44
Wheelchair	15 (1621)	15, 16
Activity did not occur	1.5 (163)	1.3, 1.8
Stair Climbing		
Up and Down Stairs Without Help	22 (2364)	22, 23
Up and Down Stairs with Help	18 (1890)	17, 19
Not go up and down stairs	60 (6332)	59, 61
Polypharmacy and Health Conditions		
Drugs		
0–2	5.7 (602)	5.3, 6.1
3–4	8.3 (876)	7.8, 8.8
5–7	22 (2364)	22, 23
≥ 8	64 (6744)	63, 65
Stroke	18 (1914)	17, 19
Congestive Heart Failure	14 (1458)	13, 14
Hypertension	66 (6956)	65, 67
Irregular Pulse	19 (2000)	18, 20
Peripheral Vascular Disease	9.2 (969)	8.6, 9.7
Chest Pain at Rest or on Exertion	4.4 (464)	4.0, 4.8
Dizziness or Lightheadedness	26 (2795)	26, 27
Edema	35 (3726)	34, 36
Shortness of Breath	33 (3533)	32, 34
Alzheimer’s	6.5 (684)	6, 7
Dementia	22 (2337)	21, 23
Multiple Sclerosis	1.5 (159)	1.3, 1.8
Parkinsonism	4.3 (456)	3.9, 4.7
Arthritis	56 (5900)	55, 57
Hip Fracture	4.4 (464)	4.0, 4.8

*95% CI* 95% Confidence Interval

### Associations with the rate of falls

Table 2 describes the adjusted associations with the rate of falls among our population-based sample of home care clients in the HNHB health region. All variables had a variance inflation factor less than 1.6, indicating that multicollinearity was not present in the final model. A sensitivity analysis was not conducted because 1 % of observations (*n* = 153) had a standardized residual greater than two.

**Table 2:** Adjusted associations with the rate of falls among home care clients in HNHB health region, Jan 1 – Mar 31, 2018 (*n* = 10,586)

	IRR	95% CI	*P* Value
Demographic Characteristics			
Male	1.00	1.00	1.00
Female	0.83	0.78, 0.89	< 0.001
Age			
< 60 (Reference)	1.00	1.00	1.00
60–69	0.88	0.77, 1.00	0.054
70–79	0.71	0.63, 0.81	< 0.001
80–89	0.70	0.61, 0.79	< 0.001
90–99	0.70	0.61, 0.80	< 0.001
≥ 100	0.57	0.37, 0.88	0.011
Marital Status			
Never Married	0.94	0.83, 1.06	0.277
Married (Reference)	1.00	1.00	1.00
Widowed	0.98	0.91, 1.05	0.520
Separated	1.25	1.05, 1.49	0.012
Divorced	1.06	0.95, 1.19	0.283
Other	1.21	0.97, 1.51	0.096
Functional Characteristics			
Cognitive Skills for Daily Decision-Making			
Independent (Reference)	1.00	1.00	1.00
Modified Independence	1.14	1.05, 1.23	0.001
Minimally Impaired	1.28	1.18, 1.40	< 0.001
Moderately Impaired	1.38	1.24, 1.54	< 0.001
Severely Impaired	1.19	1.04, 1.37	0.013
Activities of Daily Living Decline	1.59	1.49, 1.69	< 0.001
Primary Modes of Locomotion (Indoors)			
No Assistive Device (Reference)	1.00	1.00	1.00
Cane	1.42	1.28, 1.59	< 0.001
Walker/Crutch	1.50	1.37, 1.63	< 0.001
Scooter	2.26	1.42, 3.71	< 0.001
Wheelchair	1.35	1.21, 1.51	< 0.001
Activity Did Not Occur	1.06	0.83, 1.37	0.626
Stair Climbing			
Up and Down Stairs No Help (Reference)	1.00	1.00	1.00
Up and Down Stairs with Help	1.22	1.10, 1.34	< 0.001
Not Go Up and Down Stairs	1.22	1.11, 1.33	< 0.001
Polypharmacy and Health Conditions			
Drugs			
0–2 (Reference)	1.00	1.00	1.00
3–4	1.21	1.03, 1.43	0.022
5–7	1.11	0.96, 1.29	0.153
≥ 8	1.21	1.05, 1.39	0.007
Stroke	0.91	0.84, 0.98	0.013
Congestive Heart Failure	0.86	0.78, 0.94	< 0.001
Hypertension	0.99	0.93, 1.06	0.840
Irregular Pulse	1.00	0.92, 1.08	0.944
Peripheral Vascular Disease	0.96	0.87, 1.07	0.489
Chest Pain/Pressure at Rest or on Exertion	1.11	0.97, 1.28	0.139
Dizziness or Lightheadedness	1.43	1.33, 1.52	< 0.001
Edema	1.01	0.94, 1.07	0.854
Shortness of Breath	0.92	0.86, 0.98	0.013
Alzheimer’s	0.80	0.70, 0.92	0.001
Dementia	1.02	0.94, 1.11	0.642
Multiple Sclerosis	1.09	0.86, 1.38	0.485
Parkinsonism	1.46	1.28, 1.67	< 0.001
Arthritis	1.04	0.98, 1.11	0.189
Hip Fracture	1.13	0.98, 1.29	0.084

*IRR* Incident Rate Ratios, *95% CI* 95% Confidence Interval

Functional characteristics had statistically significant associations with the rate of falls. In particular, declines in activities of daily living were associated with an increased rate of falls (IRR = 1.59, 95% CI 1.49, 1.69; *p* <  0.001). The use of assistive devices for locomotion indoors also had statistically significant associations with the rate of falls among our sample: scooter (IRR = 2.26, 95% CI 1.42, 3.71; *p* <  0.001), walker or crutch (IRR = 1.50, 95% CI 1.37, 1.63; *p* <  0.001), cane (IRR = 1.42, 95% CI 1.28, 1.59; *p* <  0.001), and wheelchair (IRR = 1.35, 95% CI 1.21, 1.51; *p* <  0.001) use were all associated with an increased rate of falls. Moderately impaired cognitive skills for daily decision-making were also associated with a 38% increase in the rate of falls (IRR = 1.38, 95% CI 1.24, 1.54; *p* <  0.001).

Polypharmacy and health conditions had statistically significant associations with the rate of falls. Home care clients who took eight or more drugs had a 21% increase in the rate of falls (IRR = 1.21, 95% CI 1.05, 1.39; *p* = 0.007), and those who experienced dizziness or lightheadedness had a 43% increase in the rate of falls (IRR = 1.43, 95% CI 1.33, 1.52; *p <* 0.001). Home care clients who have parkinsonism had a 46% increase in the rate of falls (IRR = 1.46, 95% CI 1.28, 1.67; *p* <  0.001).

### Sex differences

Table 3 describes important differences between males and females observed within functional characteristics. The distribution of age between males and females in our population-based sample is comparable, and so the differences found are attributable to sex, rather than to age. Males who used assistive devices had a higher rate of falls compared to females who used assistive devices for locomotion indoors. For example, males who used a walker or crutch had a 61% increase in the rate of falls (IRR = 1.61, 95% CI 1.60, 1.67; *p* <  0.001), whereas females had a 43% increase (IRR = 1.43, 95% CI 1.31, 1.45; *p* = 0.050). Males who used a cane had a 60% increase in the rate of falls (IRR = 1.60, 95% CI 1.60, 1.67; *p* <  0.001), compared to females who had a 28% increase (IRR = 1.28, 95% CI 1.23, 1.31; *p* = 0.039).

**Table 3:** Adjusted sex differences among male and female home care clients

	IRR	95% CI	*P* Value
Functional Characteristics			
Cognitive Skills for Daily Decision-Making – Minimally Impaired			
Males	1.42	1.40, 1.47	< 0.001
Females	1.17	1.16, 1.18	0.035
Cognitive Skills for Daily Decision-Making – Moderately Impaired			
Males	1.41	1.40, 1.47	< 0.001
Females	1.39	1.25, 1.40	0.560
Activities of Daily Living Decline			
Males	1.54	1.52, 1.68	< 0.001
Females	1.58	1.55, 1.67	0.945
Primary Modes of Locomotion Indoors – Cane			
Males	1.60	1.58, 1.67	< 0.001
Females	1.28	1.23, 1.31	0.039
Primary Modes of Locomotion Indoors – Walker/Crutch			
Males	1.61	1.60, 1.67	< 0.001
Females	1.43	1.31, 1.45	0.050
Primary Modes of Locomotion Indoors – Wheelchair			
Males	1.47	1.44, 1.53	< 0.001
Females	1.30	1.29, 1.31	0.275
Health Conditions			
Stroke			
Males	0.82	0.78, 0.92	0.001
Females	0.97	0.92, 1.00	0.032
Congestive Heart Failure			
Males	0.75	0.70, 0.83	< 0.001
Females	0.94	0.89, 1.00	0.012
Dizziness or Lightheadedness			
Males	1.45	1.44, 1.56	< 0.001
Females	1.38	1.32, 1.43	0.517
Parkinsonism			
Males	1.53	1.32, 1.69	< 0.001
Females	1.28	1.10, 1.53	0.280

*IRR* Incident Rate Ratios, *95% CI* 95% Confidence Interval

Differences between males and females were also observed within neurological and cardiovascular health conditions. Specifically, males with these health conditions had a decrease in the rate of falls compared to females with the same conditions. For example, males who had a stroke had an 18% decrease in the rate of falls (IRR = 0.82, 95% CI 0.78, 0.92; *p* <  0.001), whereas females had a 3 % decrease (IRR = 0.97, 95% CI 0.92, 1.00; *p* = 0.032). Males with congestive heart failure had a 25% decrease in the rate of falls (IRR = 0.75, 95% CI 0.70, 0.83; *p* <  0.001), whereas females had a 4 % decrease (IRR = 0.94, 95% CI 0.89, 1.00; *p* = 0.012).

### Subgroup analyses

Tables 4, 5 and 6 (available as online appendices) describe the subgroup analyses of health conditions that were statistically significant (*p* <  0.001) in the final, adjusted model (i.e., parkinsonism, dizziness and/or lightheadedness, and congestive heart failure). Among home care clients with parkinsonism, the use of a cane was associated with a 129% increase in the rate of falls, compared to home care clients with parkinsonism who did not use an assistive device (IRR = 2.29, 95% CI 1.37, 3.86; *p* = 0.001). Conversely, the rate of falls among home care clients who do not have a parkinsonism diagnosis and use a cane for locomotion indoors was 39% higher (IRR = 1.39, 95% CI 1.24, 1.56; *p* <  0.001). There were also differences between home care clients with parkinsonism and the number of drugs they took and rates of falls. Home care clients with parkinsonism who took eight or more drugs had a 177% increase in the rate of falls (IRR = 2.77, 95% CI 1.13, 6.96; *p* = 0.027), compared to those who do not have parkinsonism (IRR = 1.18, 95% CI 1.03, 1.36; *p* = 0.021). In the subgroup analyses of home care clients who experienced dizziness and/or lightheadedness and have congestive heart failure, the findings of these analyses were similar to those who did not experience dizziness and/or lightheadedness and congestive heart failure.

## Discussion

### Principal findings

We investigated the associations with person-level characteristics and the rate of falls among home care clients using routinely collected data in Ontario, Canada. Declines in activities of daily living, the use of assistive devices (i.e., scooter, walker/crutch, cane, and wheelchair), impaired cognitive skills for daily decision-making, parkinsonism, and experiencing dizziness or lightheadedness were all associated with an increased rate of falls. Males who used assistive devices for mobility indoors had higher rates of falls compared to females, but men with neurological and cardiovascular health conditions had a decrease in the rate of falls compared to females. Home care clients with parkinsonism who used a cane indoors had a 129% increase in the rate of falls compared to those with parkinsonism who do not use an assistive device. Home care clients with parkinsonism who also took eight or more drugs had a 177% increase in the rate of falls compared to those who do not have parkinsonism.

Our findings confirm many of our hypotheses and are aligned with the existing literature describing accidental falls, assistive devices, and home care [31–34]. The increased rate of falls among health conditions (e.g., dizziness or lightheadedness, parkinsonism, etc.) was expected because these health conditions can cause individuals to be unstable on their feet and result in falls. The increased rate of falls attributed to assistive device use was an unexpected finding, given that we hypothesized the association between impaired cognitive skills for daily decision-making would have been higher. The decreased rate of falls among those who have had a stroke, live with congestive heart failure, shortness of breath, or Alzheimer’s was expected because these individuals are less mobile or bed-ridden because of the pathology of these conditions, which decreases the likelihood of falling. Our findings are consistent with previous studies identifying an association between parkinsonism and falls [10, 35] and between multiple sclerosis, wheelchair use, and falls [36]. Our findings are also generalizable to the literature on home care and supporting older adults in their home because as more Canadian older adults are homebound [37], the likelihood of falls in the home increases. Understanding the associations with rates of falls among older adults in the home is important for identifying ways in which falls can be prevented to support healthy aging in the home and avert unnecessary emergency department use attributed to injuries. We additionally identified how the risk of cane use for locomotion indoors for increasing the rate of falls differs substantially between males and females and among home care clients with and without parkinsonism, which we believe is an important finding for clinicians, home health care practitioners (e.g., personal support works, nurses, etc.), and informal caregivers (e.g., family members, friends, etc.). This information will help the care team identify subgroups of home care clients who may be at increased risk for multiple falls and implement strategies to prevent them.

### Implications for policies and practices pertaining to home care

Our findings underscore the importance of monitoring home care clients with a neurological health condition and who use an assistive device for locomotion indoors. Research on the use of a cane and gait changes among older adults with and without Alzheimer’s disease found that learning to use this assistive device required increased cognition and resulted in poorer gait performance [38]. Previous studies identified people with multiple sclerosis or who use a wheelchair or scooter for locomotion indoors to be susceptible to fall, including sustaining injuries as a result of falling [36]. These findings identify that assistive device use might precipitate falling among home care clients with a neurological health condition, and these findings are relevant to the work of individuals tasked with coordinating home care and home health care practitioners to help prevent accidental falls among higher risk patient groups. The use of assistive devices for locomotion indoors, such as canes and walkers, by home care clients is typically a supportive measure to prevent falls, and previous studies have identified that falls occurred when clients were not using these assistive devices [39]. The use of canes and/or walkers may also be attributed to the fact that these users may be weaker than non-users, and so these users may be more susceptible to falls. Individuals responsible for coordinating home care and home health care practitioners should be aware of assistive device use for locomotion and discuss and monitor safe use the use of the device with the client and other informal caregivers to limit the possibility of accidental falls in the home.

Our findings are also relevant to clinicians and policymakers in the areas of patient safety and quality improvement as these relate to home care. Specifically, our identification of the statistically significant associations between assistive device use for locomotion indoors and the rate of falls supports the idea of implementing interventions that reduce frailty and the occurrence of falls through exercise programs. A systematic review examining community-based exercise interventions found that these interventions are valuable for reducing the incidence of falls when these interventions focus on improving balance and include functional and resistance exercises [40]. A randomized controlled trial from Norway on exercise programs also found positive results with respect to improving physical health-related quality of life [41]. These findings demonstrate the value of exercise interventions for home care clients to reduce the incidence of accidental falls and improve patient safety in home care settings.

### Strengths and limitations

Our research is novel because we conducted a comprehensive, explanatory analysis of the associations with person-level characteristics with falls among home care clients in a population-based sample. We also identified strong, statistically significant associations between multiple assistive devices for locomotion indoors and falls. Our findings are strengthened by our large sample size and statistical power.

There are limitations to our research. First, our research is descriptive, rather than analytic. As such, a temporal sequence identifying whether assistive device occurred before or after the first occurrence of an accidental fall could not be determined, and this also limits the ability to make causal claims about assistive device use and the rate of falls in the home care setting. Second, we could not determine where in the home the fall occurred (e.g., fall down the stairs; fall from standing; fall in the bedroom, kitchen, washroom, etc.), which affects decisions pertaining to in-home environmental adjustments to reduce or eliminate falls. Third, our subgroup analysis of home care clients with a parkinsonism diagnosis is underpowered.

## Conclusion

Declines in activities of daily living, the use of assistive devices for locomotion indoors, impaired cognitive skills for daily decision-making, parkinsonism, and experiencing dizziness or lightheadedness are important associations with rate of falls among home care clients in Ontario, Canada. Future research could investigate, compare, and contrast the use assistive devices for locomotion outdoors and falls frequency among home care clients in other jurisdictions.

## Data Availability

The data analyzed in this study are not publicly available due to privacy and confidentiality restrictions pertaining to person-level health information, which contains personal identifiers, in Ontario, Canada; however, the data set creation plan and underlying analytic code are available from the corresponding author on reasonable request.

## Appendix

**Table 4:** Subgroup analysis of home care clients with and without Parkinsonism in HNHB health region, Jan 1-Mar 31, 2018

	Has Parkinsonism (*n* = 456)			Does Not Have Parkinsonism (*n* = 10,103)		
	IRR	95% CI	*P* Value	IRR	95% CI	*P* Value
Demographic Characteristics						
Male	1.00	1.00	1.00	1.00	1.00	1.00
Female	0.69	0.53, 0.91	0.001	0.84	0.78, 0.89	< 0.001
Age						
< 60	1.00	1.00	1.00	1.00	1.00	1.00
60–69	1.67	0.58, 4.73	0.282	0.87	0.76, 0.99	0.034
70–79	1.13	0.39, 3.19	0.801	0.72	0.63, 0.82	< 0.001
80–89	1.25	0.44, 3.50	0.637	0.69	0.61, 0.79	< 0.001
90–99	0.87	0.29, 2.60	0.794	0.70	0.60, 0.80	< 0.001
≥ 100	0	0	0	0.57	0.37, 0.88	0.011
Marital Status						
Never Married	1.05	0.62, 1.81	0.868	0.94	0.83, 1.07	0.340
Married	1.00	1.00	1.00	1.00	1.00	1.00
Widowed	1.00	0.72, 1.38	0.979	0.98	0.90, 1.05	0.535
Separated	1.11	0.39, 3.44	0.832	1.25	1.05, 1.50	0.012
Divorced	0.86	0.51, 1.45	0.570	1.08	0.96, 1.21	0.203
Other	1.18	0.35, 4.66	0.768	1.23	0.98, 1.54	0.075
Functional Characteristics						
Cognitive Skills for Daily Decision-Making						
Independent	1.00	1.00	1.00	1.00	1.00	1.00
Modified Independence	1.09	0.75, 1.58	0.626	1.14	1.05, 1.50	0.001
Minimally Impaired	1.45	0.97, 2.16	0.053	1.28	1.17, 1.40	< 0.001
Moderately Impaired	1.27	0.78, 2.07	0.315	1.39	1.24, 1.55	< 0.001
Severely Impaired	0.96	0.54, 1.71	0.893	1.20	1.04, 1.39	0.012
Activities of Daily Living Decline	1.20	0.88, 1.63	0.236	1.61	1.51, 1.72	< 0.001
Primary Modes of Locomotion (Indoors)						
No Assistive Device	1.00	1.00	1.00	1.00	1.00	1.00
Cane	2.29	1.37, 3.86	0.001	1.39	1.24, 1.56	< 0.001
Walker/Crutch	1.90	1.28, 2.83	0.001	1.47	1.35, 1.61	< 0.001
Scooter	0.88	0.16, 5.25	0.880	2.43	1.49, 4.07	< 0.001
Wheelchair	1.60	0.98, 2.60	0.055	1.34	1.20, 1.50	< 0.001
Activity Did Not Occur	0.14	0.00, 0.88	0.077	1.09	0.84, 1.41	0.500
Stair Climbing						
Up and Down Stairs No Help	1.00	1.00	1.00	1.00	1.00	1.00
Up and Down Stairs with Help	1.31	0.81, 2.11	0.250	1.21	1.09, 1.34	< 0.001
Not Go Up and Down Stairs	1.16	0.73, 1.82	0.521	1.23	1.12, 1.34	< 0.001
Polypharmacy and Health Conditions						
Drugs						
0–2	1.00	1.00	1.00	1.00	1.00	1.00
3–4	3.53	1.34, 9.56	0.010	1.15	0.98, 1.36	0.093
5–7	2.37	0.94, 6.05	0.067	1.09	0.94, 1.26	0.253
≥ 8	2.77	1.13, 6.96	0.027	1.18	1.03, 1.36	0.021
Stroke (CVA)	0.58	0.40, 0.85	0.005	0.92	0.85, 1.00	0.045
Congestive Heart Failure (CHF)	0.85	0.52, 1.63	0.487	0.86	0.79, 0.95	0.001
Hypertension (HTN)	0.91	0.70, 1.18	0.462	1.00	0.94, 1.07	0.950
Irregular Pulse	1.11	0.78, 1.60	0.561	0.99	0.91, 1.07	0.739
Peripheral Vascular Disease (PVD)	0.92	0.53, 1.63	0.762	0.97	0.87, 1.07	0.535
Chest Pain/Pressure at Rest or on Exertion	0.75	0.34, 1.74	0.489	1.13	0.98, 1.30	0.094
Dizziness or Lightheadedness	1.09	0.84, 1.42	0.518	1.45	1.35, 1.55	< 0.001
Edema	0.73	0.56, 1.63	0.025	1.02	0.96	0.542
Shortness of Breath	0.99	0.74, 1.33	0.947	0.91	0.85, 0.98	0.011
Alzheimer’s	0.81	0.44, 1.52	0.487	0.81	0.70, 0.93	0.002
Dementia	0.97	0.70, 1.34	0.843	1.03	0.94, 1.12	0.569
Multiple Sclerosis (MS)	2.27	0.59, 9.92	0.186	1.10	0.86, 1.39	0.452
Arthritis	0.93	0.72, 1.21	0.593	1.05	0.98, 1.12	0.143
Hip Fracture	0.77	0.40, 1.51	0.419	1.15	1.00, 1.32	0.057

*IRR* Incident Rate Ratios, *95% CI* 95% Confidence Interval

**Table 5:** Subgroup analysis of home care clients with and without dizziness or lightheadedness in the HNHB health region, Jan 1-Mar 31, 2018

	Has Dizziness or Lightheadedness (*n* = 2795)			Does Not Have Dizziness or Lightheadedness (*n* = 7791)		
	IRR	95% CI	*P* Value	IRR	95% CI	*P* Value
Demographic Characteristics						
Male	1.00	1.00	1.00	1.00	1.00	1.00
Female	0.80	0.72, 0.90	< 0.001	0.84	0.78, 0.91	< 0.001
Age						
< 60	1.00	1.00	1.00	1.00	1.00	1.00
60–69	0.85	0.68, 1.07	0.164	0.91	0.77, 1.07	0.243
70–79	0.61	0.49, 0.76	< 0.001	0.78	0.67, 0.92	0.002
80–89	0.56	0.45, 0.70	< 0.001	0.77	0.66, 0.90	0.001
90–99	0.58	0.45, 0.74	< 0.001	0.75	0.63, 0.89	0.001
≥ 100	0.90	0.38, 2.40	0.824	0.50	0.30, 0.84	0.008
Marital Status						
Never Married	1.11	0.89, 1.39	0.365	0.88	0.76, 1.02	0.097
Married	1.00	1.00	1.00	1.00	1.00	1.00
Widowed	1.02	0.90, 1.16	0.744	0.96	0.88, 1.05	0.366
Separated	1.24	0.91, 1.72	0.176	1.26	1.02, 1.55	0.296
Divorced	1.05	0.87, 1.27	0.621	1.06	0.92, 1.22	0.386
Other	1.59	1.11, 2.31	0.012	1.03	0.78, 1.37	0.842
Functional Characteristics						
Cognitive Skills for Daily Decision-Making						
Independent	1.00	1.00	1.00	1.00	1.00	1.00
Modified Independence	1.12	0.97, 1.28	0.110	1.17	1.06, 1.29	0.001
Minimally Impaired	1.23	1.05, 1.43	0.008	1.33	1.20, 1.48	< 0.001
Moderately Impaired	1.28	1.04, 1.58	0.017	1.45	1.27, 1.65	< 0.001
Severely Impaired	1.55	1.14, 2.10	0.004	1.16	0.99, 1.36	0.065
Activities of Daily Living Decline	1.41	1.25, 1.60	< 0.001	1.65	1.53, 1.79	< 0.001
Primary Modes of Locomotion (Indoors)						
No Assistive Device	1.00	1.00	1.00	1.00	1.00	1.00
Cane	1.38	1.15, 1.66	< 0.001	1.45	1.27, 1.67	< 0.001
Walker/Crutch	1.46	1.25, 1.60	< 0.001	1.51	1.36, 1.67	< 0.001
Scooter	2.12	1.00, 4.83	< 0.052	2.37	1.32, 4.43	0.004
Wheelchair	1.62	1.32, 1.99	< 0.001	1.28	1.12, 1.45	< 0.001
Activity Did Not Occur	0.54	0.27, 1.08	0.081	1.16	0.88, 1.54	0.282
Stair Climbing						
Up and Down Stairs No Help	1.00	1.00	1.00	1.00	1.00	1.00
Up and Down Stairs with Help	1.19	1.00, 1.41	0.057	1.22	1.08, 1.38	0.001
Not Go Up and Down Stairs	1.20	1.03, 1.41	0.023	1.23	1.10, 1.37	< 0.001
Polypharmacy and Health Conditions						
Drugs						
0–2	1.00	1.00	1.00	1.00	1.00	1.00
3–4	1.21	0.85, 1.72	0.292	1.19	0.99, 1.44	0.064
5–7	1.18	0.86, 1.61	0.301	1.07	0.91, 1.26	0.435
≥ 8	1.23	0.91, 1.67	0.174	1.18	1.01, 1.39	0.038
Stroke (CVA)	0.88	0.77, 1.00	0.053	0.92	0.83, 1.01	0.069
Congestive Heart Failure (CHF)	0.92	0.79, 1.06	0.246	0.84	0.75, 0.94	0.002
Hypertension (HTN)	0.95	0.85, 1.07	0.428	1.00	0.93, 1.09	0.951
Irregular Pulse	1.08	0.95, 1.23	0.217	0.95	0.86, 1.05	0.314
Peripheral Vascular Disease (PVD)	0.96	0.81, 1.14	0.661	0.97	0.85, 1.10	0.608
Chest Pain/Pressure at Rest or on Exertion	1.01	0.84, 1.21	0.937	1.26	1.01, 1.57	0.037
Edema	0.95	0.86, 1.06	0.403	1.02	0.95, 0.86	0.566
Shortness of Breath	0.93	0.84, 1.04	0.217	1.26	1.01, 1.57	0.018
Alzheimer’s	0.91	0.70, 1.18	0.467	0.75	0.64, 0.87	< 0.001
Dementia	1.15	0.99, 1.34	0.071	0.96	0.87, 1.06	0.395
Multiple Sclerosis (MS)	1.18	0.77, 1.83	0.453	1.05	0.80, 1.40	0.175
Parkinsonism	1.22	0.98, 1.53	0.074	1.59	1.35, 1.88	< 0.001
Arthritis	0.97	0.87, 1.09	0.637	1.07	0.99, 1.15	0.090
Hip Fracture	0.89	0.68, 1.15	0.357	1.22	1.04, 1.44	0.014

*IRR* Incident Rate Ratios, *95% CI* 95% Confidence Interval

**Table 6:** Subgroup analysis of home care clients with and without congestive heart failure in the HNHB health region, Jan 1-Mar 31, 2018

	Has Congestive Heart Failure (*n* = 1458)			Does Not Have Congestive Heart Failure (*n* = 9128)		
	IRR	95% CI	*P* Value	IRR	95% CI	*P* Value
Demographic Characteristics						
Male	1.00	1.00	1.00	1.00	1.00	1.00
Female	0.96	0.81, 1.10	0.643	0.81	0.76, 0.87	< 0.001
Age						
< 60	1.00	1.00	1.00	1.00	1.00	1.00
60–69	1.00	0.61, 1.60	0.999	0.87	0.76, 1.00	0.055
70–79	0.63	0.39, 1.00	0.053	0.73	0.64, 0.84	< 0.001
80–89	0.67	0.42, 1.10	0.098	0.71	0.62, 0.81	< 0.001
90–99	0.76	0.47, 1.20	0.269	0.69	0.59, 0.80	< 0.001
≥ 100	0.28	0.06, 1.10	0.074	0.64	0.40, 1.01	0.056
Marital Status						
Never Married	0.88	0.60, 1.30	0.533	0.94	0.83, 1.07	0.376
Married	1.00	1.00	1.00	1.00	1.00	1.00
Widowed	0.89	0.74, 1.10	0.239	0.99	0.91, 1.07	0.745
Separated	0.91	0.57, 1.40	0.675	1.31	1.09, 1.59	0.004
Divorced	1.04	0.78, 1.40	0.792	1.06	0.94, 1.20	0.316
Other	0.74	0.33, 1.70	0.462	1.25	1.00, 1.58	0.055
Functional Characteristics						
Cognitive Skills for Daily Decision-Making						
Independent	1.00	1.00	1.00	1.00	1.00	1.00
Modified Independence	1.05	0.87, 1.30	0.610	1.15	1.05, 1.25	0.001
Minimally Impaired	1.30	1.04, 1.60	0.020	1.27	1.16, 1.40	< 0.001
Moderately Impaired	1.25	0.92, 1.70	0.148	1.38	1.23, 1.56	< 0.001
Severely Impaired	1.22	0.82, 1.80	0.338	1.19	1.03, 1.39	0.018
Activities of Daily Living Decline	1.66	1.38, 2.00	< 0.001	1.58	1.47, 1.69	< 0.001
Primary Modes of Locomotion (Indoors)						
No Assistive Device	1.00	1.00	1.00	1.00	1.00	1.00
Cane	1.33	0.96, 1.80	0.089	1.43	1.27, 1.61	< 0.001
Walker/Crutch	1.26	0.97, 1.60	0.080	1.52	1.39, 1.67	< 0.001
Scooter	2.25	0.88, 6.30	0.094	2.20	1.29, 3.88	< 0.001
Wheelchair	1.47	1.08, 2.00	0.015	1.31	1.17, 1.47	< 0.001
Activity Did Not Occur	0.98	0.53, 1.80	0.959	1.07	0.81, 1.41	0.640
Stair Climbing						
Up and Down Stairs No Help	1.00	1.00	1.00	1.00	1.00	1.00
Up and Down Stairs with Help	1.11	0.83, 1.50	0.470	1.23	1.11, 1.37	< 0.001
Not Go Up and Down Stairs	1.15	0.88, 1.50	0.306	1.23	1.12, 1.35	< 0.001
Polypharmacy and Health Conditions						
Drugs						
0–2	1.00	1.00	1.00	1.00	1.00	1.00
3–4	1.80	0.63, 5.40	0.280	1.21	1.02, 1.43	0.028
5–7	1.48	0.59, 4.00	0.409	1.12	0.97, 1.30	0.128
≥ 8	1.93	0.79, 5.00	0.156	1.21	1.04, 1.39	0.010
Stroke (CVA)	0.94	0.78, 1.10	0.483	0.90	0.83, 0.98	0.014
Hypertension (HTN)	1.13	0.93, 1.40	0.216	0.98	0.91, 1.05	0.486
Irregular Pulse	0.86	0.74, 1.00	0.067	1.03	0.95, 1.13	0.443
Peripheral Vascular Disease (PVD)	1.00	0.82, 1.20	0.969	0.95	0.84, 1.07	0.379
Chest Pain/Pressure at Rest or on Exertion	1.04	0.80, 1.30	0.788	1.12	0.95, 1.32	0.185
Dizziness or Lightheadedness	1.47	1.25, 1.70	< 0.001	1.41	1.31, 1.52	< 0.001
Edema	1.10	0.94, 1.30	0.244	1.00	0.93, 1.07	0.985
Shortness of Breath	0.88	0.75, 1.00	0.138	0.92	0.86, 0.99	0.032
Alzheimer’s	1.26	0.86, 1.90	0.228	0.77	0.66, 0.89	< 0.001
Dementia	1.16	0.93, 1.50	0.187	1.01	0.92, 1.10	0.905
Multiple Sclerosis (MS)	0.48	0.88, 1.20	0.265	1.12	0.89, 1.43	0.337
Parkinsonism	1.22	0.80, 1.90	0.358	1.49	1.29, 1.71	< 0.001
Arthritis	1.04	0.88, 1.20	0.633	1.04	0.97, 1.11	0.246
Hip Fracture	1.01	0.70, 1.50	0.976	1.16	1.00, 1.34	0.052

*IRR* Incident Rate Ratios, *95% CI* 95% Confidence Interval

## Abbreviations

95% CI: 95% Confidence Interval
HNHB: Hamilton, Niagara, Haldimand, and Brant
IRR: Incident Rate Ratio
RAI-HC: Resident Assessment Instrument – Home Care

## References

[1] Kuspinar A, Hirdes JP, Berg K, McArthur C, Morris JN (2019). Development and validation of an algorithm to assess risk of first-time falling among home care clients. BMC Geriatr.

[2] Public Health Agency of Canada (2014). Seniors’ falls in Canada.

[3] Isaranuwatchai W, Perdrizet J, Markle-Reid M, Hoch JS (2017). Cost-effectiveness analysis of a multifactorial fall prevention intervention in older home care clients at risk for falling. BMC Geriatr.

[4] Sinn C-LJ, Betini RSD, Wright J, Eckler L, Chang BW, Hogeveen S (2018). Adverse events in home care: identifying and responding with interRAI scales and clinical assessment protocols. Can J Aging.

[5] Byers AL, Sheeran T, Mlodzianowski AE, Meyers BS, Nassisi P, Bruce ML (2008). Depression and risk for adverse falls in older home health care patients. Res Gerontol Nurs.

[6] Bjerk M, Brovold T, Skelton DA, Bergland A (2018). Associations between health-related quality of life, physical function and fear of falling in older fallers receiving home care. BMC Geriatr.

[7] Lavis JN, Hammill AC, Lavis JN (2017). Care by sector. Ontario’s health system: key insights for engaged citizens, professionals and policymakers.

[8] Guthrie DM, Harman LE, Barbera L, Burge F, Lawson B, McGrail K (2019). Quality Indicator rates for seriously ill home care clients: analysis of resident assessment instrument for home care data in six Canadian provinces. J Palliat Med.

[9] Shaw J, Bastawrous M, Burns S, McKay S. System issues leading to “found-on-floor” incidents: a multi-incident analysis. J Patient Saf. 2016. 10.1097/PTS.0000000000000294. [Epub ahead of print] 10.1097/PTS.0000000000000294 27811588

[10] Bansal S, Hirdes JP, Maxwell CJ, Papaioannou A, Giangregorio LM (2016). Identifying fallers among home care clients with dementia and Parkinson’s disease. Can J Aging.

[11] Mondor L, Maxwell CJ, Bronskill SE, Gruneir A, Wodchis WP (2016). The relative impact of chronic conditions and multimorbidity on health-related quality of life in Ontario long-stay home care clients. Qual Life Res.

[12] Poss JW, Sinn C-LJ, Grinchenko G, Blums J, Peirce T, Hirdes J (2017). Location, location, location: characteristics and Services of Long-Stay Home Care Recipients in retirement homes compared to others in private homes and long-term care homes. Healthc Policy.

[13] Schluter PJ, Ahuriri-Driscoll A, Anderson TJ, Beere P, Brown J, Dalrymple-Alford J (2016). Comprehensive clinical assessment of home-based older persons within New Zealand: an epidemiological profile of a national cross-section. Aust N Z J Public Health.

[14] Jamieson HA, Nishtala PS, Scrase R, Deely JM, Abey-Nesbit R, Connolly MJ (2018). Drug burden and its association with falls among older adults in New Zealand: a National Population Cross-Sectional Study. Drugs Aging.

[15] Doran D, Hirdes JP, Blais R, Baker GR, Poss JW, Li X (2013). Adverse events associated with hospitalization or detected through the RAI-HC assessment among Canadian home care clients. Healthc Policy.

[16] Mitchell LA, Hirdes J, Poss JW, Slegers-Boyd C, Caldarelli H, Martin L (2015). Informal caregivers of clients with neurological conditions: profiles, patterns and risk factors for distress from a home care prevalence study. BMC Health Serv Res.

[17] Mofina AM, Guthrie DM (2014). A comparison of home care quality indicator rates in two Canadian provinces. BMC Health Serv Res.

[18] Vu M, Hogan DB, Patten SB, Jette N, Bronskill SE, Heckman G (2014). A comprehensive profile of the sociodemographic, psychosocial and health characteristics of Ontario home care clients with dementia. Chronic Dis Inj Can.

[19] Harrell FE (2001). Regression modeling strategies: with applications to linear models, logistic regression, and survival analysis.

[20] Fox J (2003). Effect displays in R for generalised linear models. J Stat Softw.

[21] Grosjean P, Ibanez F (2018). pastecs: Package for Analysis of Space-Time Ecological Series.

[22] R Core Team (2019). R: a language and environment for statistical computing. R Foundation for Statistical Computing.

[23] Robinson D, Hayes A (2019). broom: Convert Statistical Analysis Objects into Tidy Tibbles.

[24] Signorell A (2019). DescTools: tools for descriptive statistics.

[25] Tang Y, Horikoshi M, Li W (2016). ggfortify: Unified Interface to Visualize Statistical Result of Popular R Packages. R J.

[26] Venables W. N., Ripley B. D. (2002). Modern Applied Statistics with S.

[27] Wickham H (2017). tidyverse: Easily install and load the ‘Tidyverse’.

[28] Wickham H (2019). modelr: Modelling Functions that Work with the Pipe.

[29] Wickham H, Hester J, Francois R (2018). readr: Read Rectangular Text Data.

[30] Hsu AT, Manuel DG, Taljaard M, Chalifoux M, Bennett C, Costa AP (2016). Algorithm for predicting death among older adults in the home care setting: study protocol for the risk evaluation for support: predictions for elder-life in the community tool (RESPECT). BMJ Open.

[31] Kanters DM, Griffith LE, Hogan DB, Richardson J, Patterson C, Raina P (2017). Assessing the measurement properties of a frailty index across the age spectrum in the Canadian longitudinal study on aging. J Epidemiol Community Health.

[32] Boffin N, Moreels S, Vanthomme K, Van Casteren V (2014). Falls among older general practice patients: a 2-year nationwide surveillance study. Fam Pract.

[33] Gianni C, Prosperini L, Jonsdottir J, Cattaneo D (2014). A systematic review of factors associated with accidental falls in people with multiple sclerosis: a meta-analytic approach. Clin Rehabil.

[34] Walsh M, Galvin R, Horgan NF (2017). Fall-related experiences of stroke survivors: a meta-ethnography. Disabil Rehabil.

[35] Poss JW, Hirdes JP (2016). Very frequent fallers and future fall injury: continuous risk among community-dwelling home care recipients. J Aging Health.

[36] Rice L, Kalron A, Berkowitz SH, Backus D, Sosnoff JJ (2017). Fall prevalence in people with multiple sclerosis who use wheelchairs and scooters. Medicine (Baltimore).

[37] Akhtar S, Loganathan M, Nowaczynski M, Sinha S, Condon A, Ewa V (2019). Aging at home: a portrait of home-based primary care across Canada. Healthc Q.

[38] Hunter SW, Divine A, Omana H, Wittich W, Hill KD, Johnson AM (2019). Effect of learning to use a mobility aid on gait and cognitive demands in people with mild to moderate Alzheimer’s disease: part I – cane. J Alzheimers Dis.

[39] Luz C, Bush T, Shen X (2017). Do canes or walkers make any difference? Non Use and Fall Injuries. Gerontologist.

[40] Sherrington C, Fairhall NJ, Wallbank GK, Tiedemann A, Michaleff ZA, Howard K, et al. Exercise for preventing falls in older people living in the community. Cochrane Database Syst Rev. 2019. 10.1002/14651858.CD012424.pub2. 10.1002/14651858.CD012424.pub2 PMC6360922 30703272

[41] Bjerk M, Brovold T, Davis JC, Skelton DA, Bergland A (2019). Health-related quality of life in home care recipients after a falls prevention intervention: a 6-month follow-up. Eur J Pub Health.

